# Local sequence determinants of two in-frame triplet deletion/duplication hotspots in the *RHD/RHCE* genes

**DOI:** 10.1186/1479-7364-6-8

**Published:** 2012-08-02

**Authors:** Jian-Min Chen, David N Cooper, Claude Férec

**Affiliations:** 1 Etablissement Français du Sang (EFS) – Bretagne and INSERM, U1078, Brest, France; 2 Institute of Medical Genetics, Cardiff University, Cardiff, CF14 4XN, UK

## 

Different types of human gene mutation can vary in size quite dramatically (e.g., single nucleotide substitutions vs. copy number variations), but what they all have in common is that their occurrence is often closely bound up with specific characteristics of the local DNA sequence environment [1]. Here, we highlight the importance of local sequence features that underlie the two in-frame triplet deletion/duplication hotspots in the *cis*-linked, highly homologous *RHD* and *RHCE* paralogs.

**Figure 1:**
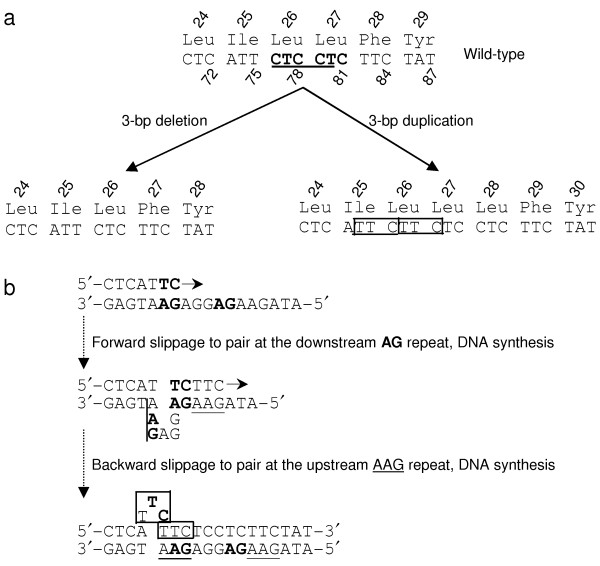
**The three currently known in-frame triplet deletion/duplication variants in exon 1 of the *RHD*/*RHCE* genes.** Their underlying mutational mechanisms are also shown. (**a**) Illustration of the identical deletion of one of two neighboring CTC repeats (*in bold*) which occurred at the corresponding positions of *RHD*[2] and *RHCE*[3] genes as well as the 3-bp in-frame duplication in the *RHD* gene [4], illustrated here as having arisen from the duplication of c.74_76 (*boxed*). The *RHD* and *RHCE* genes share 100% sequence identity in the region from c.70_87. The *underlined* CTCCT motif corresponds to the complementary strand of the DNA polymerase arrest site WGGAG. (**b**) Schema for how the 3-bp duplication could have been generated in accordance with the model of serial replication slippage (see Chen et al. [8] for details). Short direct repeats that could have mediated the two steps of replication slippage are highlighted in *bold* or are *underlined*. The *horizontal arrows* indicate the direction of DNA synthesis. The identical 3-bp deletions are explicable by a single step of forward slippage (not shown).

The first hotspot refers to an 8-bp sequence tract in exon 1 of the *RHD* and *RHCE* genes, in which three different variants were reported (Figure 1a) [2-4]. The first variant is a deletion of one of two juxtaposed CTC triplets in the *RHD* gene, which gives rise to an in-frame deletion of a single amino acid, Leu27 [2]. The second variant is identical to the first but occurred at the analogous location in the *RHCE* gene [3]. Henceforth, we shall employ the term ‘deduplication’ [5], which emphasizes the identity of the deleted sequence and the sequence immediately abutting the site of the deletion, to describe this particular type of microdeletion (<21 bp in length in accordance with Ball et al. [6]). Deduplication accounts for a significant proportion of disease-causing microdeletions; indeed, for microdeletion events of 2–5 bp, 38 % were found to be deduplications [6]. Replication slippage is currently regarded as the major mechanism underlying the generation of deduplications: the primer strand containing the newly synthesized first direct repeat dissociates from the template strand and then misaligns (slipping forward) at the second direct repeat; continued DNA synthesis then leads to the deletion of one of the two direct repeats. It should be pointed out that while direct repeats are a prerequisite for replication slippage, they are certainly not the sole determinant of this mutational mechanism. In this regard, we noted that the two CTC triplets together constitute a DNA polymerase arrest site (consensus sequence WGGAG, where W = A or T [7]) (Figure 1a). It is therefore tempting to speculate that a combination of these two sequence features could have served to make this short region particularly prone to replication slippage.

Recently, Pereira and colleagues reported the first in-frame triplet duplication in the *RHD* gene; this duplication affected the same short region as the aforementioned two deduplications in exon 1 (Figure 1a) [4]. As pointed out by the original authors, this duplication could have resulted from either a duplication of c.74-76TTC or c.75_77TCT. These authors emphasized the importance of a DNA motif (i.e., TTCTC that was identified by analogy to previously reported deletion-predisposing DNA motifs in the *RHD* gene [9]) in generating this duplication but did not provide a model to explain how this duplication could have been generated. Given that the sequence tract in question is prone to replication slippage, we surmised that this duplication might also be explicable in terms of such a mechanism. Indeed, as illustrated in Figure 1b, it can be readily explained by the model of serial replication slippage [8], invoking one step of forward slippage and one step of backward slippage.

**Figure 2:**
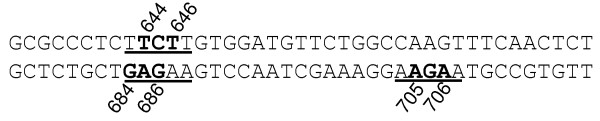
**The three 3-bp deletion variants in exon 5 of *RHD*.** The deletions are highlighted in *bold*. The *underlined* sequences refer to the consensus motif RAGAA or its complement. The c.685_687AGA deletion in RHCE [10] is not shown.

The second hotspot refers to a 63-bp region of exon 5 in the *RHD* and *RHCE* genes, in which four in-frame triplet deletions (c.644_646delTCT [3], c.684_686delGAG, and c.705_707delAGA [9] in *RHD*; c.685_687delAGA [10] in *RHCE*) were reported (Figure 2). Several distinct DNA repeats or motifs (e.g., GAGAA and GAAGA) have previously been implicated in the generation of three of these four variants [9]. A comparative evaluation of the four variants led us to propose a consensus motif RAGAA (R = A or G) (Figure 2). Since only the c.644_646delTCT variant can be explained in terms of replication slippage, it may be that RAGAA is associated with a recombination-predisposing activity that is distinct from the DNA polymerase arrest site WGGAG. In other words, the different local sequence contexts in exons 1 and 5 of *RHD* and *RHCE* could predispose to subtly different mutational processes.
